# Optimized Triton X-114 assisted lipopolysaccharide (LPS) removal method reveals the immunomodulatory effect of food proteins

**DOI:** 10.1371/journal.pone.0173778

**Published:** 2017-03-29

**Authors:** Malgorzata Teodorowicz, Olaf Perdijk, Iris Verhoek, Coen Govers, Huub F. J. Savelkoul, Yongfu Tang, Harry Wichers, Kerensa Broersen

**Affiliations:** 1 Department of Cell Biology and Immunology, Wageningen University and Research, Wageningen, the Netherlands; 2 Nanobiophysics Group, Faculty of Science and Technology, MIRA Institute for Biomedical Technology and Technical Medicine, University of Twente, Enschede, the Netherlands; 3 Food and Biobased Research, Wageningen University and Research, Wageningen, the Netherlands; Instituto Butantan, BRAZIL

## Abstract

**Scope:**

Investigations into the immunological response of proteins is often masked by lipopolysaccharide (LPS) contamination. We report an optimized Triton X-114 (TX-114) based LPS extraction method for β-lactoglobulin (BLG) and soy protein extract suitable for cell-based immunological assays.

**Methods and results:**

Optimization of an existing TX-114 based phase LPS extraction method resulted in >99% reduction of LPS levels. However, remaining TX-114 was found to interfere with LPS and protein concentration assays and decreased viability of THP-1 macrophages and HEK-Blue 293 cells. Upon screening a range of TX-114 extraction procedures, TX-114-binding beads were found to most effectively lower TX-114 levels without affecting protein structural properties. LPS-purified proteins showed reduced capacity to activate TLR4 compared to non-treated proteins. LPS-purified BLG did not induce secretion of pro-inflammatory cytokines from THP-1 macrophages, as non-treated protein did, showing that LPS contamination masks the immunomodulatory effect of BLG. Both HEK293 cells expressing TLR4 and differentiated THP-1 macrophages were shown as a relevant model to screen the protein preparations for biological effects of LPS contamination.

**Conclusion:**

The reported TX-114 assisted LPS-removal from protein preparations followed by bead based removal of TX-114 allows evaluation of natively folded protein preparations for their immunological potential in cell-based studies.

## Introduction

Increasing food demand creates a need to search for more sustainable food systems. The number of studies focused on the functionality, processing, and industrial application of alternative proteins is rapidly increasing [1]. Nutritional and immunological aspects of these novel proteins are not well-known and introducing them to the human diet requires insight into these aspects [1, 2]. *In vitro* cell culture models are a useful strategy to study the nutritional value and immunological potential of novel proteins [2, 3]. LPS (lipopolysaccharide) is a major endotoxin found in food protein preparations and other food extracts such as polysaccharides [4]. LPS is a pathogen associated molecular pattern found in the outer membrane of most gram-negative bacteria and is capable of initiating a strong innate immune response upon bacterial infection in humans [5–7]. Soluble LPS particles form a complex with lipopolysaccharide-binding protein (LBP), which is transferred to CD14 and subsequently interacts with Toll-like receptor (TLR)4 and MD-2 to activate the NF-κB pathway. This activation results in the secretion of pro-inflammatory cytokines, like IL-1β, IL-6 and TNF-α [5, 6]. Investigation of the effect of food-derived proteins on the immune response is therefore masked by high levels of immunomodulatory LPS. Removal of LPS from protein preparations reveals the unbiased immunomodulatory effects that proteins may induce. A vast array of LPS extraction procedures have been published over the years and these methods often include several chromatographic steps, such as ion exchange, hydrophobic interaction chromatography, and gel filtration [8–10]. These methods are laborious, and do not fully eliminate biological activity of residing LPS [11]. Polymyxin B (PMB) is an antibiotic that has been used as alternative strategy to eliminate LPS activity and even though PMB was shown to prevent TLR4 signaling [12] its presence does not prevent IL-1β secretion in monocyte cultures [12]. Other compounds which have been experimentally shown to eliminate the immunogenic potential of LPS include paeonol [13] and gedunin [12] although their mode of action potentially interferes with the immunogenic properties of proteins under investigation. Proanthocyanins isolated from different food sources [14] and lactoferrin [15] have also been identified as potent binding partners of LPS. However, the activity of LPS removal agents that act by interaction with LPS is influenced by the chemical properties of the protein, often require pH adjustment and do not guarantee complete dissociation and removal of protein-bound LPS [8–10]. A technique that triggers the dissociation of LPS from the protein irrespective of protein chemical properties is a two-phase detergent-based (e.g. Triton X-114, TX-114) extraction. Recently, this method was described as an efficient way of endotoxin removal from protein preparations of animal origin [16], recombinant proteins and antibodies [17–19], plasmids [20] and viral proteins [21]. TX-114 is an aqueous surfactant that assists LPS into forming micelles, which subsequently aggregate into a surfactant enriched phase at a temperature of 22°C. After phase separation, the lipophilic LPS-rich fraction can be separated from the hydrophilic protein phase by means of centrifugation [10, 16]. Although this method was shown to retain the biological activity and the structure of proteins [22, 23], TX-114 was also reported to be toxic at low concentrations to cells in culture [16] urging elimination of this extraction agent from protein preparations to allow unbiased investigation of the immunogenic potential of proteins.

In this study we demonstrate that LPS and TX-114 can be effectively removed from protein preparations to levels that do not interfere with cell viability, LPS and protein concentration assays, and *in vitro* immunological read-outs and do not impact on protein structure. We further show that LPS- and TX-114 purified β-lactoglobulin does not induce secretion of pro-inflammatory cytokines from THP-1 macrophages.

## Materials and methods

### Proteins

Bovine β-lactoglobulin (BLG) (Sigma, cat. # L0130) was dissolved in sterile phosphate buffered saline (PBS, Life Technologies, cat. # 18912–014) or MQ H_2_O. Soy protein extract (SPE) was obtained by extracting the proteins from soy flour (Sigma Aldrich, cat. # S9633) according to the procedure described by L’Hocine et al [24]. Protein concentration was determined by absorbance at 280 nm using a NanoDrop ND1000 spectrophotometer.

### Triton X-114-assisted LPS-extraction method

A previously published TX-114 based LPS extraction protocols [25, 26] was modified to optimize for LPS removal and removal of remaining TX-114. TX-114 (Amresco, cat. # M114) was added to the protein solution (BLG at 10 mg/ml and SPE at 14 mg/ml in PBS) to a final TX-114 concentration of 2% v/v. The solution was incubated at 4°C for 30 min with constant stirring. Subsequently, the sample was transferred to a water bath set at 37°C and incubated for 10 min followed by centrifugation at 20,000 g for 20 min at 37°C. The upper part containing the protein was separated from the TX-114 layer by means of pipetting and the LPS concentration was determined. To investigate whether repeated TX-114-assisted extraction increased LPS removal efficiency, the extraction procedure was repeated one, two and three times. Bio-Beads SM-2 (Bio-Rad, cat. # 152–8920) [27] with high affinity for Triton were added to the collected supernatant and incubated overnight at 4°C with constant stirring. The ratio of beads to protein solution was calculated based on the assumption that 1 g of beads adsorbs 0.07g of Triton. Using sedimentation the Bio-Beads were removed from the samples. A similarly TX-114 treated protein free buffer control was included to determine TX-114 remaining concentrations using absorption at 280 nm.

### TX-114 removal

Several approaches were evaluated for their ability to eliminate TX-114 from protein preparations. First, 8 ml protein solution was subjected to dialysis for 24 h against distilled water using a Slide-A-Lyzer 10kDa cassette (Life Technologies, cat. # 66380). Second, a volume of 1 ml of 0.1% TX-114 in buffer and 1 ml of 1.0 mg/ml of BLG were applied to a HiTrap Desalting column (GE Healthcare Life Sciences, cat. # 29-0486-84). Third, samples were centrifuged at 21,000 g and 25°C for 10 and 20 min and at 37°C for 10 and 20 min. Fourth, samples were centrifuged using 0.22 μm (cat. # 8160) and 0.45 μm (cat. # 8162) spin X filter columns (Costar) at 10,000 g at 37°C for 4 min. Last, high-affinity Triton binding Bio-Beads SM-2 (Bio-Rad, cat. # 152–8920) were tested for their efficacy to remove Triton and used according to the manufacturer manual.

### Measurement of LPS concentration

The LPS concentration was measured using the commercially available Endozyme Recombinant Factor C assay (Hyglos, cat. # 609050) according to the protocol of the manufacturer. The measurements were conducted in triplicate using a Tecan Infinite 200Pro plate reader. The positive control (MQ) was spiked with a concentration of 0.45 EU/l of LPS, and results for all tests were considered valid when the value of recovered LPS concentration was between 50 and 200% of this value. LPS units were converted from EU (Endotoxin Unit) to concentrations in pg/ml by assuming that 1 EU corresponds to 100 pg of standard endotoxin EC-5 [28]. It should be kept in mind that LPS concentration strongly depends on the source of the endotoxin, since endotoxin activity is highly variable, depending on the bacterial strain.

### SDS-PAGE

Non-reducing SDS-PAGE was used to evaluate the influence of the LPS removal procedure on protein content and quality. Samples were loaded onto a 12.5% polyacrylamide gel followed by staining using GelCode Blue Stain Reagent (Life Technologies, cat. # 24592).

### Far-UV circular dichroism

Protein samples (BLG, SPE) at 0.1 mg/ml in PBS were placed in a quartz cuvette with an optical path of 0.1 cm. Far-UV circular dichroism (CD) spectra were recorded in a Jasco J-1500 spectropolarimeter at 25°C. The wavelength range was set from 260 to 190 nm with 0.5 nm resolution, 4.0 sec response time, and 1.0 nm band width. Data were collected as averages of eight scans at a scanning speed of 50 nm/min. Spectra were corrected by subtracting the buffer baseline. Measurements were performed as independent duplicates.

### Intrinsic tryptophan fluorescence

Emission fluorescence spectra of protein samples (BLG, SPE) at 0.01 mg/ml in PBS were recorded at 25°C using a Varian Cary Eclipse spectrophotometer. The excitation wavelength was set at 295 nm (5 nm bandwidth) and the emission intensity was recorded from 310 to 500 nm (5 nm band width) at a scan rate of 120 nm/min and a 0.5 nm data interval. Spectra were corrected for buffer and represent an average of two scans. Measurements were performed as independent duplicates.

### Cell culture

HEK-Blue-hTLR4 and HEK-Blue-hTLR2 cells (InvivoGen hkb-htlr4 and hkb-htlr2) were obtained by co-transfection of the human TLR4/TLR2, MD-2 and CD14 co-receptor genes and an inducible secreted embryonic alkaline phosphatase (SEAP) reporter gene into HEK293 cells. Stimulation of TLR4 or TLR2 with LPS activates NF-κB and AP-1 (activator protein 1), which induce the production of SEAP. SEAP activity was determined with the commercially available Quanti-Blue assay (InvivoGen, cat. # rep-qb1). The cells were grown in DMEM, 4.5 g/l glucose, 10% (v/v) fetal bovine serum, 50 U/ml penicillin, 50 mg/ml streptomycin, 100 mg/ml Normocin, 2 mM L-glutamine at 37°C in a humidified atmosphere at 5% CO_2_. For the cytotoxicity and activation of TLR4 and TLR2 receptors assays cells were plated at a density of 1.25x10^3^ cells per well in a 96-well plate and incubated for 24 h. Next day the medium was replaced by 150 μl of medium containing SPE or BLG. The human monocytic leukemia cell line THP-1 (American Type Culture Collection, Rockville, Md.) was grown as described previously [29]. Macrophage differentiation was induced by treating THP-1 monocytes (10^6^ cells/ml) for 48h with 100 ng/ml phorbol 12-myristate 13-acetate (PMA, cat. # P1585) in 96-well cell culture plates containing 100 μl of cell suspension or 24-wells cell culture plates containing 500 μl of cell suspension in each well. It has been demonstrated that this differentiation method of THP-1 cells results in the expression of macrophage specific surface markers CD11b and CD36 and also phagocytic activity [30, 31].

### Cytotoxicity assay

The cytotoxic effect of TX-114 was determined using a cell viability test (CellTiter 96 AQueous One Solution Cell Proliferation Assay, Promega, cat. # G3580). HEK-Blue-hTLR4 and HEK-Blue-hTLR2 cells were incubated with 2, 1, 0.5, 0.25, and 0.13 mg/ml of BLG or 2.8, 1.4, 0.7, 0.35, and 0.175 mg/ml of SPE. Phosphate buffered saline (PBS), PBS spiked with 0.005% of TX-114 (PBS/0.005% triton) and 0.1% of TX-114 were used as controls. After 24h incubation of both cell lines a volume of 20 μL of CellTiter 96 AQueous One Solution Reagent was added to each well followed by 1 h of incubation at 37°C. Absorbance was measured at 485 nm with FilterMax F5 Multi-Mode microplate readers and the percentage of viable cells was calculated in relation to cells cultured in pure medium. All experiments were performed in triplicate.

### Activation of TLR4 and TLR2 receptors

HEK-Blue-hTLR4 and HEK-Blue-hTLR2 cells were incubated with 2, 1, 0.5, 0.25, and 0.13 mg/ml of BLG or 2.8, 1.4, 0.7, 0.35, and 0.175 mg/ml of SPE. The LPS standard curve, MQ water and MQ water spiked with 0.005% of Triton X-114 were used as controls. After 24h incubation in the presence of the stimuli the supernatant was collected and SEAP activity was measured using the Quanti-Blue assay according to the protocol of the manufacturer. The absorbance was measured at 620 nm after 1 h of the incubation at 37°C. All samples were measured in triplicate. TLR4/TLR2 receptor stimulation was expressed relative to the level of SEAP activity of cells cultured in pure medium.

### Cytokine secretion by THP1 derived macrophages

Differentiated, adherent THP-1 macrophages were washed once with sterile PBS and once with complete RPMI 1640 medium. Cells were co-cultured for 24 h with 100 μl of non-purified and LPS-purified BLG at protein concentrations of 100, 25 and 6.25 μg/ml. After 24h of incubation the supernatant was collected and human cytokine concentrations (IL-6, IL-8, IL-1β and TNF-α) were determined using the cytometric bead array (CBA) kit (Human Inflammatory Cytokine Kit, BD Bioscience, cat. # 551811) according to the manufacturer’s instructions. The samples were analyzed by flow cytometry (BD FACS Canto II, BD Bioscience). The results were normalized to cytokine levels of unstimulated macrophages cultured in the medium.

### Gene expression analysis using Q-PCR

THP-1 macrophages were incubated with titrated amounts of LPS (10^−15^ to 10^−5^ g/ml E.coli 0111:B4; Sigma) for 6 h. Cells were washed once with PBS and lysed using 200 μl TRIzol per well. RNA isolation, cDNA synthesis and RT-qPCR were performed as reported previously [30]. Investigated genes and used primers were IL-1β (FWD: GTGGCAATGAGGATGACTTGTTC; REV: TAGTGGTGGTCGGAGATTCGTA), IL-8 (FWD: CTGATTTCTGCAGCTCTGTG; REV: GGGTGGAAAGGTTTGGAGTATG), IL-10 (FWD: GTGATGCCCCAAGCTGAGA; REV: CACGGCCTTGCTCTTGTTTT), IL-12p40 (FWD: CTCTGGCAAAACCCTGACC; REV: GCTTAGAACCTCGCCTCCTT), TNFα (FWD: TTCTGCCTGCTGCACTTTG; REV: GGGTTCGAGAAGATGATCTG) and NF-κB (FWD: TGAGTCCTGCTCCTTCCA; REV: GCTTCGGTGTAGCCCATT). Relative fold-changes to non-stimulated THP-1 macrophages were calculated following normalization to GAPDH (FWD: TGCACCACCAACTGCTTAGC; REV: GGCATGGACTGTGGTCATGAG) and using the 2^-ΔΔCt^ method.

### Statistics

Results were expressed as mean ± SD of three to five independent measurements. Statistical analysis was carried out by GraphPad Prism 4 software. One-way ANOVA test with Tukey post-hoc (p<0.05, shown as one asterisk) was used to evaluate the significance if not specified differently in Fig legend.

## Results

### Triton X-114 needs to be removed from protein preparations before in vitro applications

**Fig 1:**
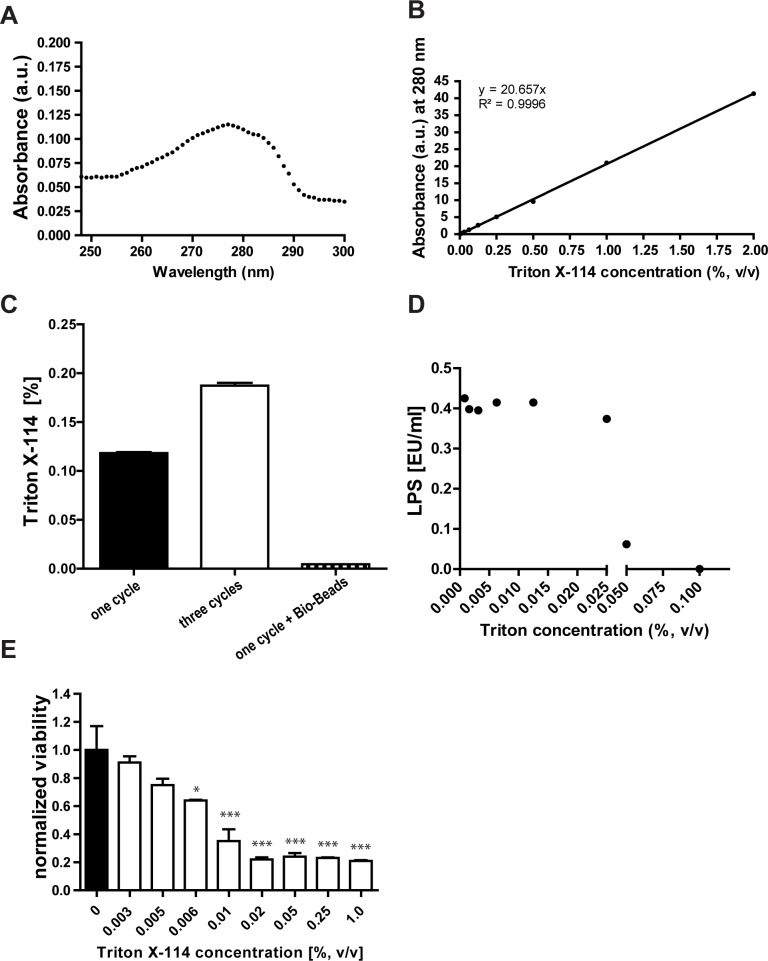
Optimization of TX-114 removal method from beta-lactoglobulin (BLG) and soy protein extract (SPE). *(A)* Triton concentrations can be quantified in protein-free solutions using spectroscopic absorbance at 280 nm. Absorbance spectrum of 0.005% (v/v) TX-114 solution in PBS was determined by a NanoDrop ND1000 spectrophotometer. *(B)* TX-114 in PBS dose-dependent absorption at 280 nm. Results are corrected for PBS background and represent an average of three independent measurements. *(C)* Concentration of TX-114 in PBS spiked with LPS (0.45 EU/l) after applying the TX-114 treatment described in Materials and Methods measured after one TX-114 cycle, three TX-114 cycles or after one TX-114 cycle followed with Bio-Beads treatment. *(D)* TX-114 reduces LPS detection with EndoZyme recombinant factor C assay in a dose-dependent manner. LPS concentration was measured in PBS spiked with 0.45 EU/l of LPS and decreasing concentration of TX-114. *(E)* Concentration of TX-114 in the medium equal or higher than 0.006% (v/v) decreases the viability of THP-1 derived macrophages. Viability of THP-1 macrophages cultured for 24 h in the presence of TX-114 in the medium expressed as relative to cells grown in TX-114 free medium = 1).

**Fig 2:**
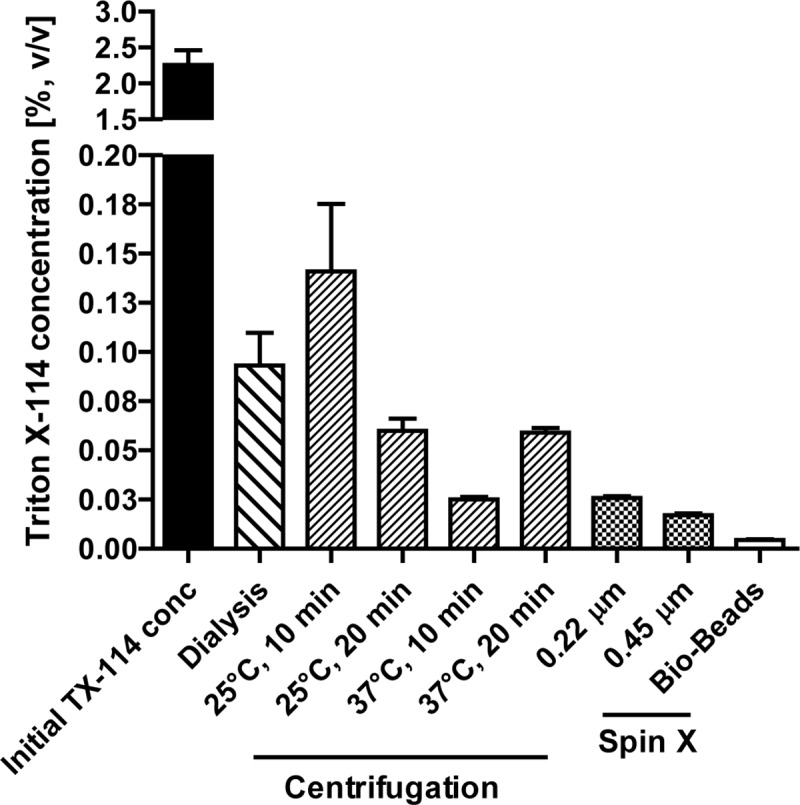
Extraction of remaining TX-114 from protein extract with high-affinity Bio-Beads results in most effective lowering of detergent concentration to non-toxic levels. Comparison of different TX-114 extraction methods. Starting from an initial TX-114 concentration of ~ 2% (v/v), the concentration of this detergent is effectively lowered by the application of dialysis, various centrifugation conditions, spin-X column and Bio-Bead assisted purification. Application of Bio-Beads results in most efficient TX-114 extraction down to 0.005% (v/v).

Even though TX-114 presents an effective LPS-extraction agent for LPS-contaminated protein preparations, residual detergent remaining in the treated protein solution has been reported to be toxic to cells in culture [16] and hamper both determination of protein by absorbing at 280 nm and LPS concentration by interfering with most commercially available LPS detection assays. These observations motivated the need to lower the remaining TX-114 concentration after the purification procedure. TX-114 concentrations were conveniently determined under conditions where no protein is present using the characteristic to absorb light at 280 nm in a linear concentration-dependent manner (Fig 1A and 1B). This allowed estimation of the concentration of TX-114 in PBS spiked with 0.45 EU/l of LPS, after applying the TX-114 treatment. After one cycle of TX-114 purification the concentation of TX-114 remaining in LPS spiked PBS was estimated to be 0.12% (v/v) and this concentration was increasing with an increasing number of purification cycles (Fig 1C). A TX-114 concentration of 0.12% (v/v) was found to interfere with the EndoZyme assay giving rise to false negative results (Fig 1D). At the same time, a concentration of TX-114 in the medium equal to or higher than 0.006% (v/v) significantly decreased the viability of THP-1 derived macrophages (Fig 1E). To evaluate the ability of a range of methods to effectively extract TX-114, PBS was spiked with 2% (v/v) TX-114 and subjected to centrifugation, dialysis, Hi-Trap desalting and spin X column purification or treatment with Triton-binding Bio-Beads (Fig 2). The use of Bio-Beads presented the most effective extraction of TX-114 by reducing TX-114 levels down to 0.005% (v/v). This concentration was significantly lower comparing to other conventional methods (Fig 2) and sufficiently low to permit reliable quantification of LPS with the EndoZyme assay (Fig 1D), UV-based determination of the protein concentration and was not cytotoxic to HEK 293 (S1 Fig) or THP-1 derived macrophages (Fig 1E).

### TX-114 assisted LPS extraction does not affect protein content and structure

**Fig 3:**
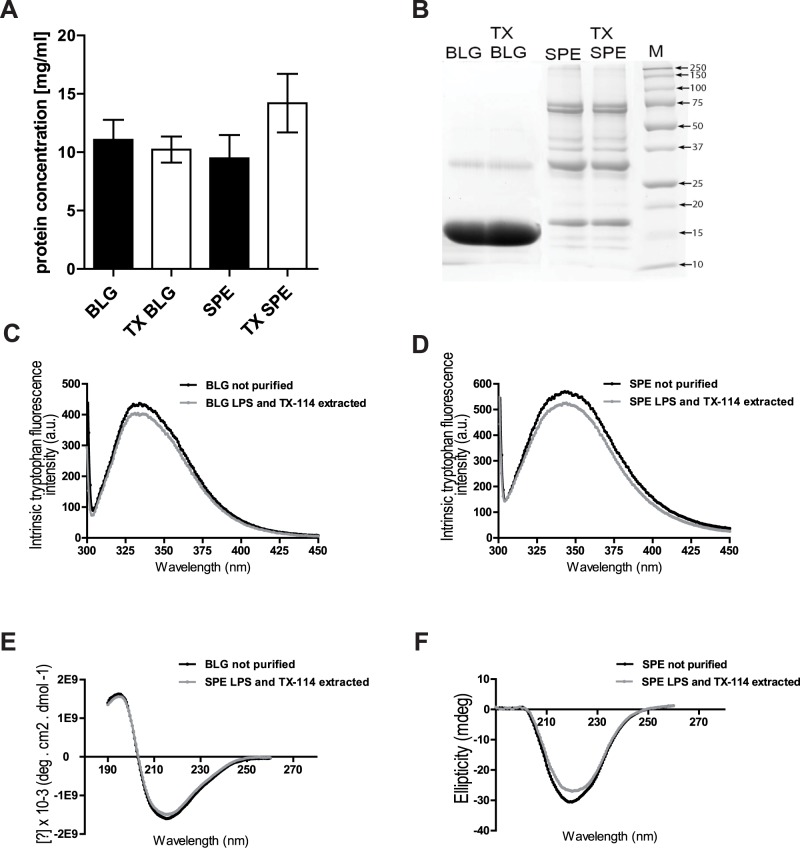
Protein yield and structure are retained upon application of the TX-114 assisted LPS extraction procedure. *(A)* BLG and SPE were dissolved in PBS at an initial concentration of ~ 10 mg/ml and protein concentrations were determined again after application of the LPS extraction procedure. (B) Non-reducing SDS-PAGE of not purified BLG, SPE and LPS- and Triton-extracted (TX BLG, TX SPE), M—molecular weight marker. *(C*, *D)* Intrinsic tryptophan fluorescence and far-UV CD spectra *(E*, *F)* of BLG and SPE. BLG concentrations used for far-UV CD were determined spectroscopically using absorbance at 280 nm and resulting CD spectra were normalized for molar ellipticity. SPE CD spectra were distorted at wavelength values below 205 nm as a result of high (>800) voltage.

TX-114-assisted LPS removal does not result in loss of proteins as protein concentrations of BLG and SPE, determined by OD280, were similar before and after application of the purification procedure (Fig 3A). Using SDS-PAGE, SPE showed a range of bands corresponding to β-conglycinin and glycinin similar to a previously reported migration profile [24]. The TX-114-assisted LPS extraction procedure did not affect this band distribution and similar results were obtained for BLG (Fig 3B) suggesting that quaternary structural arrangements were not affectd by the treatment. Further, the LPS removal procedure did not affect the secondary and tertiary structural organizations estimated from Far-UV CD and intrinsic tryptophan fluorescence (Fig 3C–3F). The CD and tryptophan fluorescence spectra largely overlap when comparing non-treated with LPS- and TX-114 extracted BLG and SPE and the wavelength of maximum tryptophan fluorescence intensity, indicative of a folded protein structure, did not differ significantly for BLG. For SPE, intrinsic tryptophan and CD peak positions for both LPS-extracted and non-treated samples were not significantly different although a small degree of variation appeared in signal intensity for both read-outs.

### LPS extraction by TX-114 significantly reduces LPS contamination of β-lactoglobulin and soy protein

**Fig 4:**
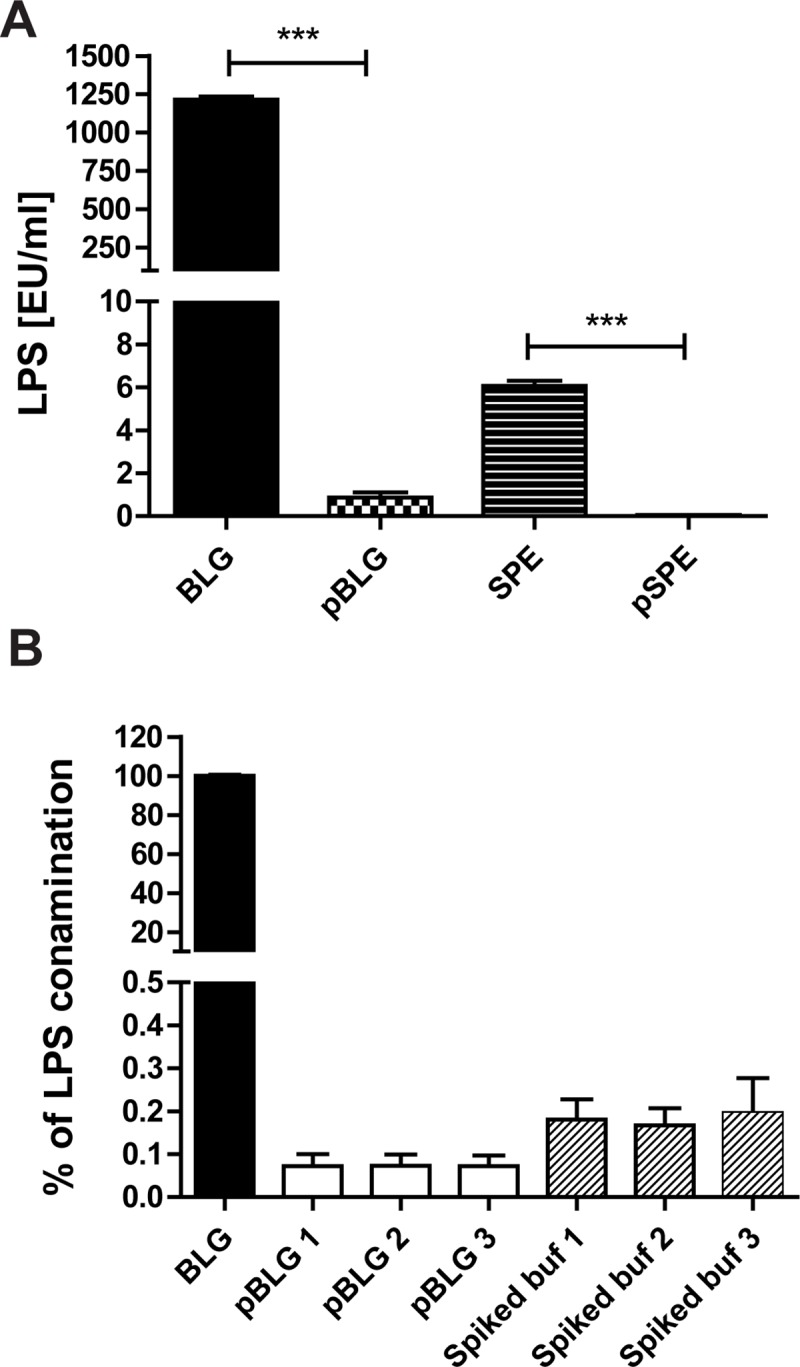
TX-114 assisted LPS extraction reduce LPS levels in BLG and SPE extracts to the levels below 1 EU/ml. *(A)* LPS concentration in protein preparations before (BLG, SPE) and after purification (pBLG, pSPE) with TX-114 method. *(B)* Repeated TX-114-assisted LPS extraction does not affect LPS extraction efficiency. BLG or PBS spiked with 0.45 EU/l of LPS were treated once, twice or three times with a 2% (v/v) TX-114 solution and subjected to incubation at 4°C for 30 min followed by a 10 min incubation at 37°C and centrifugation.

The initial concentration of LPS in BLG and SPE was estimated to be 1216 EU/ml and 6 EU/ml, respectively. TX-114-assisted LPS extraction reduced the LPS concentration in BLG to 0.87 EU/ml and in SPE to 0.05 EU/ml demonstrating in purification efficiencies of 99.9% and 99.2%, respectively (Fig 4A). Repeated (one, two or three times) LPS-extraction did not lower LPS levels further (Fig 4B) but caused an increase of the residual TX-114 concentration in the protein sample (Fig 1E).

### Stimulation of TLR4 and TLR2 receptors by β-lactoglobulin and soy protein

**Fig 5:**
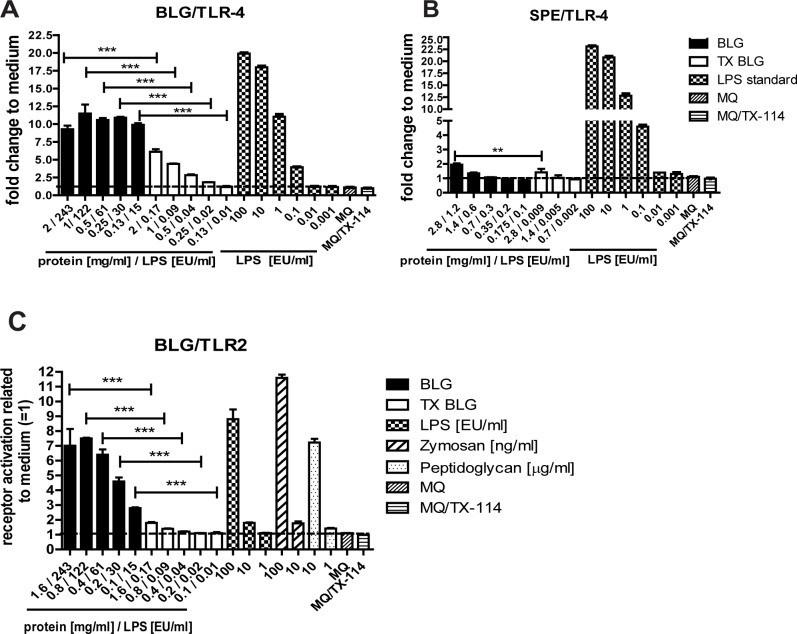
**Reduced TLR4 (A,B) and TLR2 (C) activation upon incubation of HEK-Blue 293 with LPS purified protein preparations.** HEK-Blue 293 cells were incubated 24 h with non-treated BLG (BLG, black bars A, C), BLG purified with TX-114 method (TX BLG, white bars A, C), non-treated SPE (SPE, black bars B), SPE purified with TX-114 method (TX SPE, white bars B). The samples were tested in a range of dilutions and the corresponding concentration of protein (mg/ml) and LPS (EU/ml) in each dilution is presented on the x axis. The results are expressed as the relative to unstimulated cells (medium control = 1). Statistically significant differences between corresponding dilutions of LPS-purified and non-treated preparations are shown (p<0.05). LPS standard curve, MQ water and MQ water spiked with 0.005% of TX-114 (MQ/TX-114) were used as controls.

Fig 5 shows the levels of TLR4 and TLR2 receptor mediated signaling in HEK 293 cells incubated with non-treated and TX-114 treated BLG and SPE. Incubation of HEK-Blue 293 cells with non-treated BLG resulted in high TLR4 activation (on average 10-fold higher than medium control) reaching the plateau for all tested concentrations (Fig 5A). The level of TLR4 stimulation was significantly lower (up to 88%), but followed a concentration-dependent pattern, upon incubation of cells with LPS-purified BLG. Incubation of non-treated SPE with HEK-Blue 293 cells resulted in low activation of TLR4 (2-fold higher than medium control). This was observed only for the highest concentration of protein at 2.8 mg/ml, (corresponding with 1.2 EU/ml of LPS) and was reduced but not eliminated by treatment with TX-114 (Fig 5C). These data suggest that SPE itself, in the absence of LPS, may also induce TLR4 activation. A dose-dependent activation of TLR2, at lower level compared to TLR4, was observed upon incubation of HEK-Blue 293 cells with non-treated BLG which was significantly reduced upon extraction of LPS from the protein (Fig 5C). An activation of TLR2 by LPS-purified BLG at the concentration of 2 mg/ml (0.17 EU/ml of LPS contamination) may be explained by an immunomodulatory effect of BLG itself as LPS at a concentration of 1 EU/ml did not induce TLR2 activation. However, contamination of the BLG preparation with other TLR2 ligands could not be excluded. Collectively, these data show that the described LPS extraction procedure sufficiently lowers LPS concentrations to reveal the immunomodulatory effects of proteins.

### THP-1 macrophages as a model to study immunomodulatory potential of BLG

**Fig 6:**
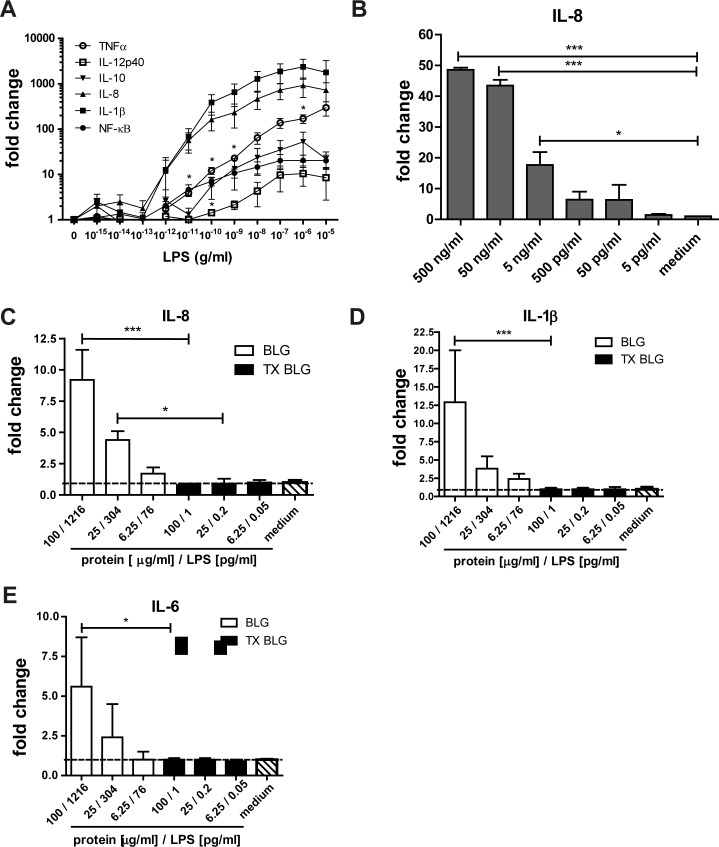
THP-1 derived macrophages as a model to study immunomodulatory potential of non-purified and LPS-purified BLG preparation. *(A)* LPS dose-dependence and differential gene expression of inflammatory cytokines and NF-κB in THP-1 macrophages. The data obtained for 3 repetitions is normalized to GAPDH and relative to unstimulated macrophages cultured in medium. Statistically significant differences relative to non-stimulated THP-1 macrophages were calculated with Student’s *t*-tests: * = P<0.05. *(B)* LPS dose-dependent secretion of IL-8 by THP-1 macrophages. THP-1 cells were incubated 24h with increasing concentration of LPS and IL-8 concentration in supernatant was determined with flow cytometry. The results and statistically significant differences are related to unstimulated cells medium control = 1). *(C-E)* Dose dependent secretion of pro-inflammatory cytokines from THP-1 macrophages incubated 24h with non-purified BLG (BLG) and BLG purified with TX-114 method (TX BLG). The results are expressed as the relative to unstimulated cells (medium control = 1). Statistically significant differences between corresponding dilutions of purified and non-purified BLG preparations are shown (p<0.05).

To establish the potential of THP-1 derived macrophages to serve as a model to study the immunogenic potential of proteins we first analyzed the sensitivity threshold of THP-1 macrophages to *E*. *Coli* derived LPS by analyzing gene expression of inflammatory cytokines and measurement of released IL-8 levels in the medium (Fig 6A and 6B). LPS concentrations of 10 pg/ml and higher induced a pro-inflammatory gene response in THP-1 macrophages by significantly increasing TNFα expression. Gene expression levels of IL-1β and IL-8 were increased albeit not significantly (Fig 6A). These observations at the mRNA level directly translated into increased levels of IL-8 upon incubation of THP-1 cells with LPS at a concentration of ≤50 pg/ml (Fig 6B). Next, we incubated THP-1 macrophages with non-treated and LPS-purified BLG to investigate its immunomodulatory potential in the absence of interfering LPS. Non-treated BLG at a concentration of 25 μg/ml (304 pg/ml of LPS) significantly increased the level of IL-8 while higher concentration of BLG of 100 μg/ml increased significantly the secretion of IL-1β and IL-6 (Fig 6C–6E). Incubation of cells with LPS-purified BLG diminished the secretion of all three cytokines (IL-1β, IL-8 and IL-6) when compared to non-treated BLG, which was shown to be contaminated with LPS. These observations indicate that LPS can be eliminated from protein preparation in such a manner that the isolated natively folded protein-induced immunogenic effects on immune cells in culture can be reliably measured in the absence of LPS.

## Discussion

Our work aimed at optimizing an LPS extraction procedure from proteins to enable functional investigation of the immunological properties of these proteins without the interfering effect of LPS or LPS extraction agents.

TX-114 based phase separation was shown to be an efficient, simple and cost-effective strategy to significantly reduce or even eliminate endotoxin contamination from biological preparations [9, 16–18, 20–23, 26, 32]. Despite the scientific need for LPS removal to establish the immunological activity of natively folded proteins, only few studies reported on the experimental conditions that affect the efficiency of endotoxin removal from protein extracts using TX-114. Reported protocols vary in incubation conditions and TX-114-removal strategies after LPS-extraction [11, 16, 17, 32], which we have shown to critically influence the application of the purified protein in *in vitro* assays [26]. Lopes *et al*. (2010) showed that separation of LPS into a TX-114 micelle-rich phase is more effective at higher TX-114 concentrations and that extraction efficiency is positively correlated with increasing incubation temperature (33–41°C) [26]. We therefore used a concentration of TX-114 of 2% (v/v) and incubated the TX-114-protein mixture at a temperature of 37°C, resulting in an LPS extraction efficiency of 99.9% for BLG and 99.2% for SPE after a single TX-114 treatment. When using TX-114 at a concentration of 1% (v/v), some studies observed improved LPS-extraction efficiency after repeated treatment of the protein solution with TX-114 [16, 17, 23]. We observed that the efficiency of LPS extraction did not increase further with additional cycles of 2% (v/v) TX-114 treatment. The observed differences may be explained by interactions between LPS and proteins resulting in the formation of protein–endotoxin complexes, as described before for different types of proteins [33, 34]. Such endotoxin-protein complexes are able to activate Toll-like receptor 4 and induce the secretion of pro-inflammatory cytokines as it has been shown previously for lactoferrin [15]. Protein associated LPS is difficult to remove during purification procedures [10] although Reichelt and colleagues showed affinity chromatography with TX-114 as an effective method to remove tightly bound endotoxin from recombinant proteins [18]. Although to our knowledge the interaction between LPS and BLG and soy proteins have not been reported but can also not be excluded. Therefore the application of optimized TX-114 (2%) for studied proteins provided sufficient detergent and micelle forming ability to dissociate also protein-bound LPS as no secretion of pro-inflammatory cytokines was observed after incubation of macrophages with purified BLG/soy protein sample. Nevertheless, the efficiency at which endotoxin is removed, especially when tightly associated, from a protein may depend on various factors including the type of interactions between the endotoxins and the protein. A number of proteins and peptides with different physicochemical characteristics [21, 34–37] were shown to interact with LPS via electrostatic interactions for basic proteins and hydrophobic interactions or formation of dynamically stable calcium bridges played a role for neutral and acidic proteins [9, 38]. Moreover it has been shown that the concentration of the protein itself effects significantly the amount of disaggregated endotoxin and the formation of protein-LPS complexes. The protein concentration was also shown to has significant effect on protein-LPS binding and endotoxin removal by ultrafiltration membranes (300,000 NMWCO) [39]. Because many variables influence the LPS extraction efficiency, it is challenging to develop one method that is generally suitable for LPS removal from different protein sources and it cannot be concluded that the method described here is indeed suitable to extract LPS from all these different proteins. However, the TX-114 method applied in this study was demonstrated to efficiently extract LPS from two proteins with highly different physico-chemical profiles including BLG, a protein of animal origin belonging to the lipocalin family and SPE, a mixture of storage proteins, mostly globulins, isolated from soy.

The protein structure-function paradigm dictates that any change in protein structure, induced by the LPS extraction procedure may directly translate into an alternative immunological profile [40]. Although the TX-114 treatment does not influence the biological activity and functionality of proteins [23, 25, 41] a limited number of studies focus on the structural properties of the proteins after treatment with TX-114 [22]. BLG and SPE studied here, did not show differences in secondary, tertiary and quaternary structure upon treatment as determined by SDS-PAGE, far-UV circular dichroism spectroscopy and intrinsic tryptophan fluorescence. Moreover, TX-114 treatment did not result in loss of BLG and SPE based on protein concentration and SDS-PAGE. However, based on literature [42], the significant decrease in protein concentration after TX-114 treatment of hydrophobic proteins can be expected making the procedure less suitable for highly hydrophobic proteins.

A major disadvantage of using TX-114 to extract LPS removal is that residual detergent persists in the protein phase after extraction resulting in toxicity of the preparations to living cells. We observed that 0.1% of TX-114 in the medium cause 35% decrease in the viability of HEK 293 cells while a concentration of 0.006% and higher was already cytotoxic for THP-1 macrophages. TX-114 concentrations of 0.02% (v/v) were previously reported to influence cellular activity of PMA-stimulated neutrophils [16] demonstrating that, even though all cell types investigated responded to TX-114, different cell types may vary in their sensitivity towards this detergent. Virtual elimination of residual TX-114 after LPS extraction therefore seems crucial for applications using cell based read-outs. We further reported that TX-114 can interfere with common protein and LPS concentration determination assays. Previously published procedures often disregard this phenomenon giving rise to conclusions based on TX-114 induced bias in concentration assays [11, 23, 26, 43]. Buetler and colleagues [11] showed that glycolaldehyde-modified BLG preparations were unable to induce inflammatory signaling in receptor for RAGE-expressing cells after TX-114 assisted extraction of LPS. The authors concluded that cell activation shown for non-treated preparations was connected to a LPS-like lipophilic contamination. Interestingly, data presented by the same authors for glycolaldehyde-modified BLG preparations purified using affinity chromatography, which similarly resulted in efficient reduction of LPS contamination, demonstrated that incubation of cells with these preparations induced expression of TNFα. Despite similar virtual elimination of LPS in both preparations, the TX-114-assisted LPS extracted glycolaldehyde-modified BLG did not induce inflammatory signaling while proteins treated by affinity chromatography did. This apparent inconsistency may be explained by the impact of TX-114 remaining after LPS-extraction in the TX-114 treated BLG. Although centrifugation was applied in this last study to remove TX-114 from BLG preparations we observed that centrifugation reduced TX-114 levels to ±0.025% (v/v), a concentration that unambiguously affects cell viability. Similarly, Jensen and colleagues [23] showed that TX-114 phase separation can be used to remove LPS from (His)6-tagged proteins and that biological activity of the treated recombinant protein was retained. These authors performed multiple cycles of TX-114 extraction followed by dialysis to remove residual TX-114 levels. However, the TX-114 concentration after dialysis was not determined, while we observed that dialysis does not sufficiently lower TX-114 concentrations to levels that do not interfere with *in vitro* and cell-assays. Above examples show the need to actively remove remaining TX-114 levels from treated protein solutions and to monitor levels of TX-114 after LPS-extraction. Based on our experiments comparing a range of TX-114 removal procedures, the application of Triton-binding Bio-Beads was found to reduce the TX-114 concentration to a non-cytotoxic concentration of 0.005% (v/v) without influencing the protein structure and yield upon treatment as previously reported [20,22,22,19]. The optimized TX-114 based LPS-extraction procedure described in this manuscript allowed investigation of the immunologic potential of protein preparations without interference of LPS and TX-114. For this, we used HEK293 cells expressing the LPS receptor subunits (TLR4, CD14 and MD-2) and THP-1 macrophages that are naturally sensitive to endotoxins. Similar to Schwarz and colleagues [44] we observed that incubation of HEK 293 cells with LPS showed activation of TLR4 and -2 at LPS concentrations higher than 1 pg/ml and 100 pg/ml, respectively. LPS at a concentration of 10 pg/ml and higher increased the expression of pro-inflammatory cytokines from THP-1 macrophages. Consistent with these findings, LPS concentrations of 10 and 20 pg/ml were reported to induce secretion of IL-6 and TNF-α from murine bone marrow-derived dendritic cells [45] and activation of monocytes isolated from human blood, especially CD1c+ dendritic cells [44]. Compatibility of the outcomes suggests that both HEK293 cells expressing the LPS receptor subunits and differentiated THP-1 macrophages may be used as a relevant model for the screening of protein preparations for biological effects of LPS contamination. The biological effects of LPS depend not only on its concentration but also on the source of endotoxin. It has been shown that the primary structure of the lipid A moiety, but also a specific conformation enhance its biological activity by enabling binding to the Toll-like receptors and accessory proteins, LPS binding protein (LBP), CD14 and the (TLR4)–MD-2 complex [6, 46, 47]. That suggests that comparable LPS amounts present in protein preparations may cause different biological effects dependently of the source of contamination. Therefore before an application in immunological *in vitro* studies each protein preparation should be tested individually in the functional screening assay for LPS activity like presented in this paper HEK 293 cell line transfected with TLR-4 receptor. Incubation of TLR4 transfected HEK293 cells with LPS-extracted BLG resulted in stimulation of TLR4 but significantly higher levels were achieved by non-treated BLG demonstrating that contaminating LPS interferes with reliable immunogenic profiling of protein preparations. Similarly, while non-treated BLG induced large-scale upregulation of IL-8, IL-1β and IL-6 in THP-1 macrophages, upon LPS-extraction of BLG this effect was eliminated. The BLG protein preparations, under these conditions and up to a concentration of 100 μg/ml, did not show immunomodulatory properties. This finding is in line with that of Brix and colleagues [48] who showed that endotoxin is a major immunostimulatory component present in commercial β-lactoglobulin preparations.

## Conclusion

Investigation of immunomodulatory properties of food proteins requires an unequivocal conclusion on the source of the observed immunological activity. We optimized an LPS extraction method consisting of a two-step procedure involving TX-114 assisted LPS extraction followed by treatment of the protein preparations using high-affinity triton-binding beads to reduce the detergent residuals to non-toxic levels. The procedure was further validated with functional assays using HEK293 cells expressing LPS receptor subunits and differentiated THP-1 macrophages to demonstrate that the TX-114 based LPS extraction method allows measurement of the immunologic potential of proteins without the interfering effect of LPS or Triton.

## Supporting information

S1 Fig Triton X-114 at a concentration of 0.005% (v/v) does not influence the viability of HEK-Blue 293 cells. HEK-Blue 293 cells were cultured for 24 h in the presence of non-treated BLG (BLG) and BLG purified with TX-114 (TX BLG) and the viability of cells was measured with CellTiter 96 AQueous One Solution Cell Proliferation Assay. Results are expressed as relative to unstimulated cells (medium control = 1). PBS, PBS spiked with 0.005% (v/v) TX-114 and 0.1% (v/v) TX-114 were used as controls.
(EPS)
